# Supplementary material for: Dynamics of Plasma Lipidome in Progression to Islet Autoimmunity and Type 1 Diabetes – Type 1 Diabetes Prediction and Prevention Study (DIPP)
Source: Sci Rep. 2018 Jul 13;8:10635. doi: 10.1038/s41598-018-28907-8 (PMC6045612; doi:10.1038/s41598-018-28907-8)
Supplement: Supplementary file 1 — Supplementary information [file 41598_2018_28907_MOESM1_ESM.docx]

# **Dynamics of Plasma Lipidome in Progression to Islet Autoimmunity and Type 1 Diabetes – Type 1 Diabetes Prediction and Prevention Study (DIPP)**

Santosh Lamichhane^1^, Linda Ahonen^2^, Thomas Sparholt Dyrlund^2^, Esko Kemppainen^1^, Heli Siljander^3^, Heikki Hyöty^4,5^, Jorma Ilonen^6,7^, Jorma Toppari^8,9^, Riitta Veijola^10,11,12^, Tuulia Hyötyläinen^13^, Mikael Knip^3,14,15^*, Matej Oresic^1,16^*

^1^Turku Centre for Biotechnology, University of Turku and Åbo Akademi University, Turku 20520, Finland.

^2^Steno Diabetes Center Copenhagen, 2820 Gentofte, Denmark

^3^Children's Hospital, University of Helsinki and Helsinki University Hospital, 00290 Helsinki, Finland; Research Program Unit, Diabetes and Obesity, University of Helsinki, 00290 Helsinki, Finland.

^4^Faculty of Medicine and Life Sciences, University of Tampere, Tampere, Finland

^5^Fimlab Laboratories, Pirkanmaa Hospital District, Tampere, Finland

^6^Immunogenetics Laboratory, Institute of Biomedicine, University of Turku, Turku, Finland

^7^Clinical Microbiology, Turku University Hospital, Turku, Finland

^8^Institute of Biomedicine, Centre for Integrative Physiology and Pharmacology, University of Turku, Turku, Finland

^9^Department of Pediatrics, Turku University Hospital, Turku, Finland

^10^Department of Paediatrics, PEDEGO Research Unit, Medical Research Centre, University of Oulu, Oulu, Finland

^11^Department of Children and Adolescents, Oulu University Hospital, Oulu, Finland

^12^Department of Women’s and Children’s Health, Karolinska Institutet, Stockholm, Sweden

^13^Department of Chemistry, Örebro University, 702 81 Örebro, Sweden

^14^Tampere Center for Child Health Research, Tampere University Hospital, Tampere, Finland

^15^Folkhälsan Research Center, Helsinki, Finland

^16^School of Medical Sciences, Örebro University, 702 81 Örebro, Sweden

*Corresponding and shared senior authors:

Mikael Knip, M.D., Ph.D.; Children's Hospital, University of Helsinki, P.O.Box 22, FI-00014 Helsinki, Finland. Phone: +358 59 4487722; Email: [mikael.knip@helsinki.fi](mailto:mikael.knip@helsinki.fi)

Matej Orešič, Ph.D. Turku Centre for Biotechnology, University of Turku and Åbo Akademi University, Tykistokatu 6, FI-20520 Turku, Finland. Phone: +358 44 972 6094; Email: [matej.oresic@utu.fi](mailto:matej.oresic@utu.fi)

**Supplementary information**

**Figure captions**

**Figure 1.** The relationship between the case/control ratio (PT1D/CTRL) and acyl chain content in lipid species. The mean ratio of lipid levels in p PT1D vs CTRL in plasma samples (ages 3 months) for phosphatidylcholines (PCs). Red circles indicate upregulated while blue circles indicate downregulated acyl chain content.

**Figure 2.** Regression coefficient plot. This plot has the regression coefficient on the x-axis and VIP scores on the y-axis. a) The positive and negative regression coefficient are representative of P1Ab and the PT1D, respectively. b) The positive and negative regression coefficient are representative of P1Ab and the CTRL, respectively. Each dot represents individual lipids, labelled based on their lipid class identity. Abbreviations: Cholesterol ester (CE), Diacylglycerol (DG), Lysophosphatidylcholine (LPC), Phosphatidylcholine (PC), Phosphatidylethanolamine (PE), Sphingomyelin (SM), and Triacylglycerol (TG).

**Figure 3.** The differences in the serum lipidome between two groups, i.e. the PT1D with IAA group vs. the P1Ab with IAA group. The horizontal bar plot highlights that most of the lipids (SMs, TGs,PC) with the exception of CE were decreased in the PT1D with IAA group when compared to the P1Ab with IAA group at the age of 3 months. Abbreviations: Cholesterol ester (CE), Phosphatidylcholine (PC), Sphingomyelin (SM), and Triacylglycerol (TG).

# Supplementary Figures

**FIGURE 1**


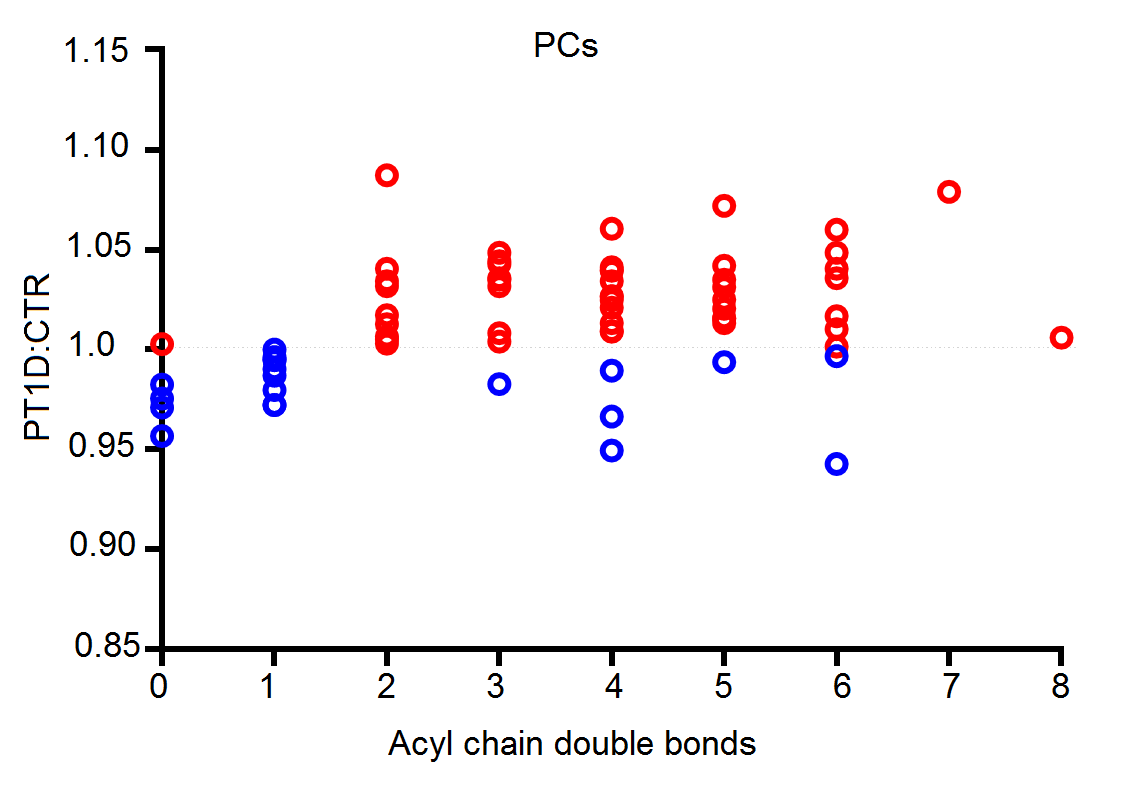


**FIGURE 2**

**
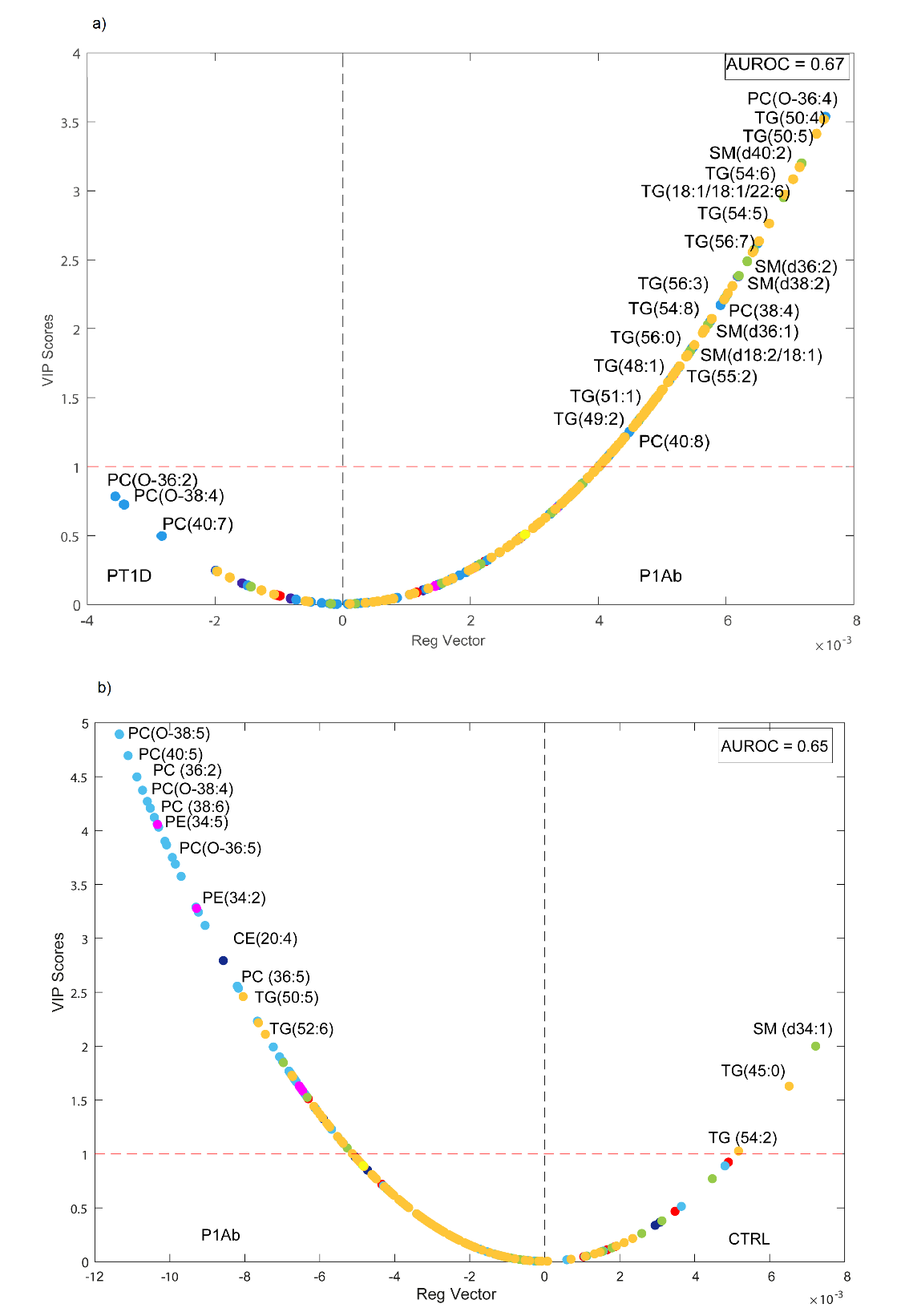
**

**FIGURE 3**

**
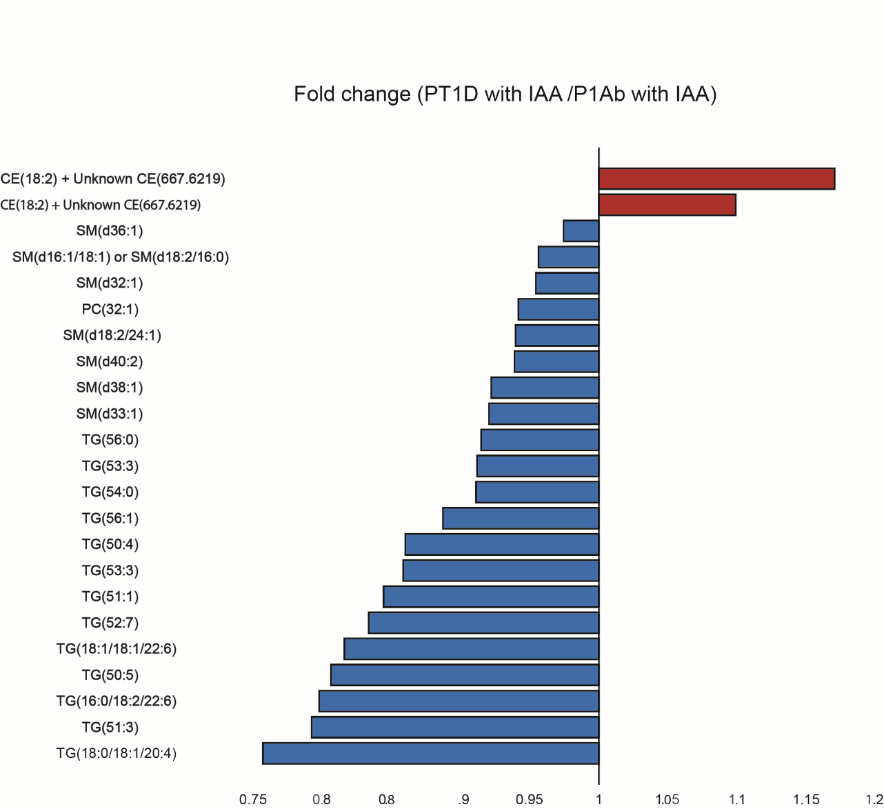
**

**Table 1:** Differential plasma lipids between CTRL and PT1D at age 3 months.

| Lipid species | P-values | q-values |
| --- | --- | --- |
| SM(d34:1) | 0.002534 | 0.151834 |
| SM(d36:1) | 0.002537 | 0.151834 |
| SM(d36:2) | 0.005078 | 0.202658 |
| PC(40:5) | 0.031458 | 0.300013 |
| PC(O-22:2/22:3) | 0.021073 | 0.300013 |
| PC(O-36:2) | 0.049273 | 0.300013 |
| PC(O-38:4) or PC(P-38:3) | 0.048787 | 0.300013 |
| SM(d18:2/18:1) | 0.04657 | 0.300013 |
| SM(d36:0) | 0.02618 | 0.300013 |
| SM(d38:0) | 0.04841 | 0.300013 |
| SM(d38:2) | 0.013263 | 0.300013 |
| TG(55:2) | 0.024865 | 0.300013 |

Abbreviations: Phosphatidylcholine (PC), Sphingomyelin (SM), and Triacylglycerol (TG).

**Table 2:** Differential plasma lipids between P1Ab and PT1D at age 3 months

| Lipid species | **p-values** | **q-values** |
| --- | --- | --- |
| **TG(50:4)** | 0.00125 | 0.03432 |
| **TG(50:5)** | 0.002515 | 0.03432 |
| **SM(d40:2)** | 0.002528 | 0.03432 |
| **TG(54:6)** | 0.004259 | 0.03432 |
| **TG(18:1/18:1/22:6)** | 0.004524 | 0.03432 |
| **PC(O-36:4)** | 0.005637 | 0.03432 |
| **SM(d16:1/18:1) or SM(d18:2/16:0)** | 0.007069 | 0.03432 |
| **TG(16:0/18:2/22:6)** | 0.007869 | 0.03432 |
| **SM(d33:1)** | 0.007875 | 0.03432 |
| **SM(d18:2/18:1)** | 0.008253 | 0.03432 |
| **TG(18:0/18:1/20:4)** | 0.008253 | 0.03432 |
| **SM(d38:2)** | 0.010805 | 0.041188 |
| **TG(54:5)** | 0.01466 | 0.043126 |
| **TG(54:5)** | 0.016194 | 0.043126 |
| **LPC(20:4)** | 0.016203 | 0.043126 |
| **PC(36:5)** | 0.017877 | 0.043126 |
| **SM(d32:1)** | 0.017877 | 0.043126 |
| **TG(56:6)** | 0.019343 | 0.043126 |
| **TG(56:3)** | 0.021312 | 0.043126 |
| **PC(31:0)** | 0.021641 | 0.043126 |
| **PC(36:4)** | 0.021685 | 0.043126 |
| **SM(d36:2)** | 0.021685 | 0.043126 |
| **TG(56:7)** | 0.021685 | 0.043126 |
| **SM(d36:1)** | 0.02384 | 0.044129 |
| **PE(38:6)** | 0.024865 | 0.044129 |
| **PC(38:4)** | 0.02613 | 0.044129 |
| **PC(18:0p/22:6)** | 0.027971 | 0.044129 |
| **TG(51:3)** | 0.028701 | 0.044129 |
| **PC(O-40:4)** | 0.028715 | 0.044129 |
| **TG(56:5)** | 0.030963 | 0.044129 |
| **TG(46:2)** | 0.031402 | 0.044129 |
| **TG(18:2/18:1/16:0)** | 0.031458 | 0.044129 |
| **TG(54:8)** | 0.03346 | 0.044129 |
| **PC(32:0)** | 0.034424 | 0.044129 |
| **PC(38:4)** | 0.034424 | 0.044129 |
| **TG(55:2)** | 0.035839 | 0.044129 |
| **TG(52:7)** | 0.036734 | 0.044129 |
| **TG(14:0/18:2/18:2)** | 0.037306 | 0.044129 |
| **TG(52:5)** | 0.037625 | 0.044129 |
| **PC(O-36:3)** | 0.041076 | 0.046946 |
| **SM(d18:0/14:0)** | 0.044792 | 0.046946 |
| **TG(49:2)** | 0.048711 | 0.046946 |
| **PC(O-38:4)** | 0.048768 | 0.046946 |
| **PC(O-36:5)** | 0.048787 | 0.046946 |
| **TG(56:7)** | 0.049273 | 0.046946 |
|  |  |  |

Abbreviations: Lysophosphatidylcholine (LPC), Phosphatidylcholine (PC), Phosphatidylethanolamine (PE), Sphingomyelin (SM), and Triacylglycerol (TG).

**Table 3:** Differential plasma lipids between CTRL and P1Ab at age 3 months

| Lipid species | **p-values** | **q-values** |
| --- | --- | --- |
| **PC(O-40:4)** | 9.23E-05 | 0.008908 |
| **PE(34:2)** | 0.000191 | 0.009199 |
| **PC(32:2)** | 0.000597 | 0.019195 |
| **PC(34:3)** | 0.000946 | 0.022805 |
| **PE(36:4)** | 0.001671 | 0.030056 |
| **PC(36:3)** | 0.002259 | 0.030056 |
| **PC(O-36:4)** | 0.002514 | 0.030056 |
| **PC(P-20:0/22:4)** | 0.003028 | 0.030056 |
| **PC(36:2)** | 0.003237 | 0.030056 |
| **PC(34:2)** | 0.003427 | 0.030056 |
| **PC(O-38:5)** | 0.003427 | 0.030056 |
| **PC(O-34:2)** | 0.00511 | 0.041079 |
| **PC(35:2)** | 0.005634 | 0.04117 |
| **PC(O-38:4)** | 0.006205 | 0.04117 |
| **PC(35:3)** | 0.006828 | 0.04117 |
| **PC(40:5)** | 0.006828 | 0.04117 |
| **PC(38:3)** | 0.007507 | 0.041838 |
| **PC(40:4)** | 0.007865 | 0.041838 |
| **PE(38:6)** | 0.008597 | 0.041838 |
| **PC(38:4)** | 0.009049 | 0.041838 |
| **PC(40:5)** | 0.009472 | 0.041838 |
| **PC(36:2)** | 0.009922 | 0.041838 |
| **PC(O-40:4)** | 0.009975 | 0.041838 |
| **PC(36:4)** | 0.010868 | 0.041938 |
| **PC(38:6)** | 0.010868 | 0.041938 |
| **PC(37:3)** | 0.012519 | 0.04645 |
| **PC(37:2)** | 0.013005 | 0.046466 |
| **PC(40:6)** | 0.014207 | 0.048947 |
| **PE(38:4)** | 0.015079 | 0.049433 |
| **PC(35:4)** | 0.015373 | 0.049433 |
| **PC(40:8)** | 0.017395 | 0.05413 |
| **PC(O-36:3)** | 0.020049 | 0.060439 |
| **TG(50:5)** | 0.023688 | 0.069245 |
| **PC(37:4)** | 0.02538 | 0.070871 |
| **PC(O-36:5)** | 0.025713 | 0.070871 |
| **TG(50:4)** | 0.027846 | 0.074618 |
| **CE(18:2)** | 0.028928 | 0.075422 |
| **PC(O-38:4)** | 0.032712 | 0.083043 |
| **PC(40:6)** | 0.034048 | 0.08359 |
| **CE(20:4)** | 0.035381 | 0.08359 |
| **PC(O-22:2/22:3)** | 0.035527 | 0.08359 |
| **PC(O-38:6)** | 0.037645 | 0.086466 |
| **PC(O-42:3)** | 0.042283 | 0.094859 |
| **PC(36:5)** | 0.044532 | 0.097634 |
| **PC(O-40:6)** | 0.047708 | 0.102273 |

Abbreviations: Cholesterol ester (CE), Phosphatidylcholine (PC), Phosphatidylethanolamine (PE), and Triacylglycerol (TG).

**Table 4:** Differential plasma lipids between B-P1Ab and A-P1Ab.

| Lipid species | p-values | q-values |
| --- | --- | --- |
| CE(20:5) | 0.000347 | 0.059825 |
| PC(36:5) | 0.001572 | 0.135339 |
| LPC(18:0) | 0.003105 | 0.172123 |
| PC(36:4) | 0.003997 | 0.172123 |
| TG(54:8) | 0.007527 | 0.181389 |
| SM(d36:1) | 0.007586 | 0.181389 |
| PC(P-20:0/22:4) | 0.009166 | 0.181389 |
| PC(40:8) | 0.009399 | 0.181389 |
| PC(32:0) | 0.010002 | 0.181389 |
| CE(20:4) | 0.011104 | 0.181389 |
| PC(38:3) | 0.011584 | 0.181389 |
| LPC(16:0e) | 0.018204 | 0.261286 |
| PC(36:4) | 0.0213 | 0.282207 |
| TG(58:10) | 0.023514 | 0.289292 |
| SM(d36:2) | 0.025392 | 0.291565 |
| PC(38:4) | 0.029813 | 0.302016 |
| TG(18:1/18:1/22:6) | 0.030791 | 0.302016 |
| LPC(20:3) | 0.033768 | 0.302016 |
| PC(40:5) | 0.034414 | 0.302016 |
| SM (d36:0) | 0.037215 | 0.302016 |
| PC(O-36:5) | 0.039297 | 0.302016 |
| TG(18:2/22:5/16:0) | 0.040326 | 0.302016 |
| TG(14:0/16:0/18:1) | 0.042866 | 0.302016 |
| TG(48:1) | 0.044816 | 0.302016 |
| SM(d32:1) | 0.044996 | 0.302016 |
| PC(38:6) | 0.04559 | 0.302016 |
| SM(d40:1) | 0.047717 | 0.3044 |

Abbreviations: Cholesterol ester (CE), Lysophosphatidylcholine (LPC), Phosphatidylcholine (PC), Sphingomyelin (SM), and Triacylglycerol (TG).

**Table 5:** Differential plasma lipids between B-PT1D and A-PT1D.

| Lipid species | p-values | q-values |
| --- | --- | --- |
| LPC(18:2)_[LVL2] | 0.002241 | 0.375457 |
| SM(d18:1/12:0)_[LVL2] | 0.004293 | 0.375457 |
| TG(57:0)_[LVL3] | 0.006736 | 0.375457 |
| CE(20:5)_[LVL2] | 0.009051 | 0.375457 |
| PC(36:2)_[LVL2] | 0.009223 | 0.375457 |
| SM(d36:1)_[LVL2] | 0.012132 | 0.378227 |
| PC(36:5)_[LVL2] | 0.013008 | 0.378227 |
| LPC(20:3)_[LVL2] | 0.016025 | 0.407722 |
| SM(d32:1)_[LVL2] | 0.021776 | 0.449747 |
| PC(O-36:2)_[LVL2] | 0.022096 | 0.449747 |
| TG(54:8)_[LVL3] | 0.033566 | 0.545093 |
| PC(38:5)_[LVL2] | 0.036198 | 0.545093 |
| Fragment: CE sigture ion_[LVL2] | 0.037394 | 0.545093 |
| SM(d36:2)_[LVL2] | 0.037493 | 0.545093 |
| PC(O-34:3)_[LVL2] | 0.043777 | 0.581595 |
| PC(O-38:6)_[LVL2] | 0.045718 | 0.581595 |

Abbreviations: Cholesterol ester (CE), Lysophosphatidylcholine (LPC), Phosphatidylcholine (PC), Sphingomyelin (SM), and Triacylglycerol (TG).

**Table 6:** Differential plasma lipids between before and after the emergence of with IAA in PT1D.

| Lipid species | p-values | q-values |
| --- | --- | --- |
| TG(57:0)_[LVL3] | 0.001156 | 0.457412 |
| SM(d18:1/12:0)_[LVL2] | 0.002954 | 0.235131 |
| PC(O-36:2)_[LVL2] | 0.012896 | 0.355229 |
| Fragment: CE sigture ion_[LVL2] | 0.014324 | 0.355229 |
| PC(O-34:2)_[LVL2] | 0.014884 | 0.355229 |
| PC(36:2)_[LVL2] | 0.01925 | 0.355229 |
| SM(d36:1)_[LVL2] | 0.019595 | 0.355229 |
| SM(d36:2)_[LVL2] | 0.019651 | 0.355229 |
| LPC(20:3)_[LVL2] | 0.020086 | 0.355229 |
| PC(O-34:3)_[LVL2] | 0.022587 | 0.35952 |
| CE(20:4)_[LVL2] | 0.027444 | 0.370544 |
| PC(34:2)_[LVL2] | 0.027935 | 0.370544 |
| PC(36:5)_[LVL2] | 0.037275 | 0.410876 |
| PC(38:5)_[LVL2] | 0.038413 | 0.410876 |
| PE(38:6)_[LVL2] | 0.041351 | 0.410876 |
| LPC(18:2)_[LVL2] | 0.0438 | 0.410876 |
| CE(18:1) + Unknown CE(669.6358)_[LVL2] | 0.046717 | 0.410876 |

Abbreviations: Cholesterol ester (CE), Lysophosphatidylcholine (LPC), Phosphatidylcholine (PC), Sphingomyelin (SM), and Triacylglycerol (TG).

**Table 7:** Differential plasma lipids between before and after the emergence of with all others except IAA in PT1D.

| Lipid specie | p-value | q-value |
| --- | --- | --- |
| LPC(18:2)_[LVL2] | 0.013222 | 0.982661 |

Abbreviation: Lysophosphatidylcholine (LPC)

**Table 8:** Differential plasma lipids between before and after the emergence of with IAA in P1Ab.

| Lipid species | p-values | q-values |
| --- | --- | --- |
| TG(54:8)_[LVL3] | 0.012228 | 0.32554 |
| CE(20:4)_[LVL2] | 0.014281 | 0.32554 |
| TG(58:10)_[LVL3] | 0.016798 | 0.32554 |
| CE(20:5)_[LVL2] | 0.017329 | 0.32554 |
| PC(40:5)_[LVL2] | 0.018152 | 0.32554 |
| TG(48:1)_[LVL3] | 0.02207 | 0.32554 |
| TG(14:0/16:0/18:1)_[LVL2] | 0.025156 | 0.32554 |
| PC(O-42:3)_[LVL2] | 0.025399 | 0.32554 |
| TG(51:0)_[LVL3] | 0.028382 | 0.32554 |
| PC(O-40:6)_[LVL2] | 0.030122 | 0.368243 |
| PC(O-38:5)_[LVL2] | 0.032424 | 0.636852 |
| TG(46:1)_[LVL3] | 0.037347 | 0.368243 |
| SM(d36:1)_[LVL2] | 0.038356 | 0.32554 |
| PC(36:5)_[LVL2] | 0.039247 | 0.32554 |
| TG(50:0)_[LVL3] | 0.039325 | 0.672949 |
| PC(30:0)_[LVL2] | 0.043434 | 0.32554 |
| TG(50:0)_[LVL2] | 0.045348 | 0.32554 |
| PC(O-36:5)_[LVL2] | 0.046562 | 0.32554 |
| PC(36:1)_[LVL2] | 0.047412 | 0.32554 |
| TG(52:7)_[LVL3] | 0.047903 | 0.32554 |
| PC(40:8)_[LVL2] | 0.04816 | 0.32554 |
| SM(d36:0)_[LVL2] | 0.049756 | 0.32554 |

Abbreviations: Cholesterol ester (CE), Phosphatidylcholine (PC), Sphingomyelin (SM), and Triacylglycerol (TG).

**Table 9:** Differential plasma lipids between before and after the emergence of with all others except IAA in P1Ab.

| Lipid species | p-values | q-values |
| --- | --- | --- |
| CE(20:5)_[LVL2] | 0.007115 | 0.745543 |
| PC(36:4)_[LVL2] | 0.007815 | 0.745543 |
| LPC(18:0)_[LVL1] | 0.013241 | 0.745543 |
| PC(36:5)_[LVL2] | 0.020014 | 0.745543 |
| PC(O-34:3)_[LVL2] | 0.020029 | 0.745543 |
| PC(P-20:0/22:4)_[LVL2] | 0.023612 | 0.745543 |
| LPC(20:3)_[LVL2] | 0.035273 | 0.745543 |
| SM(d36:2)_[LVL2] | 0.043205 | 0.745543 |
| PC(38:3)_[LVL2] | 0.044525 | 0.745543 |
| PE(36:4)_[LVL2] | 0.044765 | 0.745543 |
| TG(54:5)_[LVL3] | 0.046813 | 0.761912 |

Abbreviations: Cholesterol ester (CE), Lysophosphatidylcholine (LPC), Phosphatidylcholine (PC), Phosphatidylethanolamine (PE), Sphingomyelin (SM), and Triacylglycerol (TG).

**Table 10:** Differential plasma lipids between PT1D with IAA (n= 8) vs P1Ab with IAA (n= 6) at 3 months of age.

| Lipid species | p-values | q-values | Fold change |
| --- | --- | --- | --- |
| SM(d33:1) | 0.007992 | 0.097039 | 0.920536 |
| SM(d32:1) | 0.012654 | 0.097039 | 0.954459 |
| TG(18:0/18:1/20:4) | 0.012654 | 0.097039 | 0.756954 |
| TG(50:4) | 0.012654 | 0.097039 | 0.860021 |
| SM(d36:1) | 0.01998 | 0.097039 | 0.974489 |
| SM(d40:2) | 0.01998 | 0.097039 | 0.938978 |
| TG(51:3) | 0.01998 | 0.097039 | 0.79232 |
| SM(d38:1) | 0.028638 | 0.097039 | 0.922167 |
| SM(d18:2/24:1) | 0.029304 | 0.097039 | 0.939727 |
| TG(16:0/18:2/22:6) | 0.029304 | 0.097039 | 0.797689 |
| TG(50:5) | 0.029304 | 0.097039 | 0.80626 |
| TG(51:1) | 0.029304 | 0.097039 | 0.844221 |
| TG(52:7) | 0.041958 | 0.097039 | 0.833533 |
| TG(53:3) | 0.041958 | 0.097039 | 0.911885 |
| TG(56:0) | 0.041958 | 0.097039 | 0.914857 |
| CE(18:2) + Unknown CE(667.6219) | 0.042624 | 0.097039 | 1.170539 |
| CE(18:2) + Unknown CE(667.6219) | 0.042624 | 0.097039 | 1.09898 |
| PC(32:1) | 0.042624 | 0.097039 | 0.941818 |
| SM(d16:1/18:1) or SM(d18:2/16:0) | 0.042624 | 0.097039 | 0.956405 |
| TG(18:1/18:1/22:6) | 0.042624 | 0.097039 | 0.815937 |
| TG(53:3) | 0.042624 | 0.097039 | 0.858501 |
| TG(54:0) | 0.042624 | 0.097039 | 0.911251 |
| TG(56:1) | 0.042624 | 0.097039 | 0.887266 |

Abbreviations: Cholesterol ester (CE), Phosphatidylcholine (PC), Sphingomyelin (SM), and Triacylglycerol (TG). Here fold change represent the value mean (PT1D with IAA (n= 8)) / mean (P1Ab with IAA (n= 6))
